# Reappraising Kidney Biopsy in Diabetic Kidney Disease: Histopathology, Clinical Course, and the Future of Precision Nephrology

**DOI:** 10.3390/jcm15145359

**Published:** 2026-07-09

**Authors:** Maja Pieczaba, Bartosz Dubniański, Zofia Kuźnik, Weronika Wajerowska, Andrzej Konieczny, Mirosław Banasik

**Affiliations:** 1 Clinical Department of Nephrology, Transplantation Medicine and Internal Diseases, Institute of Internal Diseases, Wroclaw Medical University, 50-556 Wroclaw, Poland; andrzej.konieczny@umw.edu.pl (A.K.); miroslaw.banasik@umw.edu.pl (M.B.); 2 Faculty of Medicine, Wroclaw Medical University, 50-367 Wroclaw, Poland; bartosz.dubnianski@student.umw.edu.pl (B.D.); weronika.wajerowska@student.umw.edu.pl (W.W.)

**Keywords:** diabetic kidney disease, diabetic nephropathy, kidney biopsy, renal biopsy, non-diabetic kidney disease, histopathology, interstitial fibrosis and tubular atrophy, precision nephrology

## Abstract

**Background**: Diabetic kidney disease (DKD) is one of the leading causes of chronic kidney disease and kidney failure worldwide. Although most patients with diabetes are diagnosed clinically on the basis of albuminuria, estimated glomerular filtration rate decline, and diabetic retinopathy, clinical parameters alone may not reliably distinguish biopsy-proven diabetic nephropathy (DN) from non-diabetic kidney disease (NDKD) or mixed lesions. The role of kidney biopsy in diabetic patients therefore remains selective rather than routine, but its diagnostic and prognostic value is increasingly relevant in precision nephrology. **Methods**: This narrative review summarizes clinicopathological evidence on kidney biopsy in patients with diabetes, with particular emphasis on the Renal Pathology Society classification of DN, the prognostic significance of glomerular, tubulointerstitial, and vascular lesions, indications for biopsy, and emerging digital and molecular approaches to renal tissue assessment. **Results:** Histopathological evaluation provides information that cannot be fully captured by routine clinical markers. While glomerular lesions remain central to the classification of DN, tubulointerstitial fibrosis and tubular atrophy are among the strongest predictors of kidney disease progression. Vascular lesions, particularly arteriolar hyalinosis, also carry renal and cardiovascular prognostic significance. Kidney biopsy is especially valuable in patients with atypical clinical features, including rapid decline in kidney function, acute-onset nephrotic-range proteinuria, active urinary sediment, short diabetes duration, absence of diabetic retinopathy, or suspicion of immune-mediated glomerular disease. In these settings, biopsy may identify NDKD or mixed DN/NDKD, with direct therapeutic implications, including the potential use of disease-specific or immunosuppressive treatment. Emerging technologies, including artificial intelligence-assisted digital pathology, spatial transcriptomics, multi-omics profiling, and biomarker-based “virtual biopsy” models, may further refine lesion quantification and risk stratification, although they currently complement rather than replace tissue-based diagnosis. **Conclusions**: Kidney biopsy remains a clinically important tool in selected patients with diabetes. Beyond confirming DN, it enables the detection of NDKD, improves prognostic stratification, and supports individualized therapeutic decision-making. A selective, indication-driven biopsy strategy should be regarded as an essential component of precision nephrology in DKD, particularly as histopathology becomes increasingly integrated with digital, molecular, and computational approaches.

## 1. Introduction

Diabetic nephropathy (DN) is a major microvascular complication of diabetes mellitus (DM) and a leading cause of chronic kidney disease (CKD) and end-stage renal disease (ESRD) worldwide [1]. DN refers to diabetes-specific histological changes whereas diabetic kidney disease (DKD) is a clinical diagnosis, classically characterized by persistent albuminuria and a gradual decline in renal function, ultimately leading to end-stage renal disease [2]. It develops in a substantial proportion of patients, affecting up to 40% of individuals with diabetes, and is particularly associated with type 2 diabetes in developed countries [3]. With the global prevalence of diabetes exceeding 460 million people and continuing to rise, DKD represents an increasing clinical and socioeconomic burden, often necessitating lifelong renal replacement therapies such as dialysis or kidney transplantation [4]. Pathophysiologically, chronic inflammation and progressive fibrosis are central mechanisms driving disease onset and progression. These mechanisms have been increasingly well characterized at the cellular level, encompassing metabolic, hemodynamic, and immune-mediated pathways in which mesangial cells, macrophages, and tubular epithelial cells act as both targets and amplifiers of glomerular and tubulointerstitial injury [5,6,7]. Despite the intensive search for non-invasive biomarkers of early kidney damage, kidney biopsy remains the only definitive method to confirm the underlying histopathological changes in DN and rule out ischemic or immunological non-diabetic kidney diseases (NDKD). This distinction is crucial, as the presence of NDKD often dictates a completely different, sometimes immunosuppressive, therapeutic approach. Nevertheless, due to its invasive nature, the risk of bleeding complications, and a lack of universal consensus on indications, routine biopsy in diabetic patients remains controversial; therefore, this review aims to critically evaluate its role, clinical utility, and prognostic value.

## 2. Pathophysiology of DKD

The pathogenesis of DKD is primarily driven by chronic hyperglycemia, which disrupts cellular metabolism and induces excessive production of reactive oxygen species (ROS) [8]. This oxidative stress damages DNA, proteins, and lipids, impairing the glomerular filtration barrier.

Hyperglycemia activates multiple metabolic pathways, including the polyol pathway, protein kinase C (PKC), advanced glycation end-products (AGEs)–receptor for AGE (RAGE) signaling, and the hexosamine pathway, which, together with renin–angiotensin–aldosterone system (RAAS) activation, promote intraglomerular hypertension, endothelial dysfunction, and inflammation [9,10,11,12]. In the polyol pathway, aldose reductase converts excess intracellular glucose to sorbitol and subsequently fructose, generating osmotic and oxidative stress that accelerates non-enzymatic cross-linking of type IV collagen and drives thickening of the glomerular basement membrane (GBM), the earliest ultrastructural lesion detectable in DKD [6,7]. AGE-RAGE signaling on mesangial cells upregulates transforming growth factor-β (TGF-β) and connective tissue growth factor, stimulating mesangial cell proliferation and excessive accumulation of mesangial matrix, which manifests histologically as mesangial expansion and, with disease progression, as Kimmelstiel–Wilson nodules [5,6]. TGF-β signaling itself subsequently exerts a dual structural effect: through Smad2/3-dependent signaling it drives podocyte cytoskeletal reorganization and epithelial–mesenchymal transition, accelerating foot-process effacement and podocyte detachment from the GBM, while its sustained activation in the tubulointerstitial compartment stimulates fibroblast proliferation and excessive extracellular matrix deposition, leading to tubulointerstitial fibrosis and tubular atrophy [5,7].

Concurrently, glomerular endothelial cells also undergo injury, characterized by glycocalyx degradation, loss of fenestrations and increased permeability. These alterations contribute to disruption of the glomerular filtration barrier and promote podocyte injury through impaired endothelial–podocyte crosstalk. Podocytes subsequently develop characteristic structural alterations, including foot-process effacement, slit diaphragm disruption, cytoskeletal rearrangement, hypertrophy, apoptosis, and eventual detachment from GBM.

Because mature podocytes lack self-renewal capacity, their structural alterations represent an irreversible step toward progressive glomerulosclerosis and the onset of albuminuria [13,14,15,16].

These pathological changes are further amplified by reciprocal interactions among mesangial cells, podocytes, and glomerular endothelial cells, creating a self-perpetuating cycle of glomerular injury and extracellular matrix deposition [16].

In addition, increasing evidence suggests that activation of the complement system, particularly via the alternative pathway, contributes to the pathogenesis of DKD by amplifying inflammatory responses, endothelial injury, and tissue fibrosis. Complement deposition within renal biopsy specimens has been associated with more advanced histopathological lesions and worse renal outcomes, suggesting that complement activation may influence both the histological phenotype and the clinical progression of diabetic nephropathy [17].

## 3. Histopathology of DKD

### 3.1. Glomerular Lesions

Histological alterations representing DN can be detected as early as two years after diabetes onset, often preceding the clinical diagnosis of DKD [18]. Initial changes include glomerular basement membrane (GBM) thickening, which precedes clinically detectable albuminuria and is modulated by podocyte activity [19]. Subsequently, mild mesangial expansion develops; however, progressive accumulation of mesangial matrix leads to the formation of Kimmelstiel–Wilson nodules and eventually global glomerulosclerosis [20].

The 2010 Renal Pathology Society (RPS) classification, commonly referred to as Tervaert’s classification, stratifies diabetic nephropathy into four hierarchical glomerular lesion classes, with separate evaluation of tubulointerstitial and vascular involvement [21]. Class I represents the earliest ultrastructural change, with isolated GBM thickening detectable only by electron microscopy. Class II is defined by mesangial expansion, subdivided into mild (IIa) and severe (IIb) forms affecting more than 25% of the mesangium but without nodular sclerosis. Class III corresponds to nodular sclerosis, marked by at least one Kimmelstiel–Wilson lesion, defined as focal, lobular, round to oval mesangial lesions with an acellular, hyaline or matrix core. Class IV indicates advanced diabetic glomerulosclerosis, characterized by global glomerulosclerosis in over 50% of glomeruli [21].

Classes of nodular lesions in DN according to RPS classification are presented in Table 1.

### 3.2. Interstitial and Tubular Lesions

Furthermore, while the RPS framework provides standardized grading for glomerular lesions, it also mandates the independent scoring of interstitial fibrosis and tubular atrophy (IFTA), recognizing these chronic changes as some of the most powerful independent predictors of eGFR decline [22,23]. In type 2 DM, severe arteriosclerosis frequently contributes to chronic ischemic injury, exacerbating tubular and interstitial fibrosis, whereas in type 1 diabetes, tubulointerstitial lesions typically follow glomerular damage [24,25]. Notably, tubulointerstitial inflammation, fibrosis, and functional impairment may occur early in type 2 DM, sometimes preceding overt glomerular pathology, highlighting the heterogeneity and prognostic importance of these lesions.

Histologically, tubulointerstitial inflammation is characterized by mononuclear inflammatory cell infiltrates within the renal interstitium, composed predominantly of macrophages and T lymphocytes, with fewer dendritic cells. These inflammatory infiltrates are commonly associated with tubular epithelial injury and become more extensive as diabetic nephropathy progresses. In advanced disease, tubulointerstitial inflammation frequently coexists with interstitial fibrosis and tubular atrophy (IFTA), which represent the major chronic tubulointerstitial lesions [23]. Among chronic tubulointerstitial lesions, IFTA represents the final common histopathological manifestation of chronic kidney injury [26,27]. The severity of IFTA observed in renal biopsy specimens is a key determinant of disease chronicity and is widely used to predict renal outcomes and guide therapeutic decisions [27]. IFTA has been identified as a critical factor influencing kidney prognosis [28], and its severity correlates strongly with disease progression in DN. Although urinary NAG and β2-microglobulin correlate with the degree of IFTA [29], adding their excretion levels to known predictors of progression does not improve prognostication, suggesting that IFTA score may be superior to these markers in predicting renal prognosis in advanced DN [30]. Notably, urinary retinol-binding protein (RBP) also correlates with IFTA severity and, unlike NAG and β2-microglobulin, has demonstrated independent prognostic value [29]. Importantly, IFTA has been identified as an independent risk factor for disease progression in type 2 diabetic nephropathy [31]. While glomerular, interstitial, and vascular lesions all contribute to declining renal function, IFTA appears to be a more reliable predictor of rapid eGFR decline than proteinuria [32]. Advanced stages of IFTA are also independently associated with both adverse renal outcomes and increased all-cause mortality [33].

### 3.3. Vascular Lesions

Arteriolar hyalinosis is a characteristic vascular lesion in DN, affecting both afferent and efferent arterioles, with involvement of the efferent arteriole considered highly indicative of diabetic etiology [34]. Although such changes may also occur with aging, they tend to develop earlier in individuals with diabetes or hypertension [35]. The presence of arteriolar hyalinosis in renal biopsy specimens has been associated with an increased risk of disease progression and poorer renal outcomes [35,36]. While not entirely specific to DN, hyalinosis of both afferent and efferent arterioles is relatively uncommon in other conditions, reinforcing its diagnostic relevance [37]. Moreover, more advanced forms of arteriolar hyalinosis have been identified as predictors of the development of macroalbuminuria and reduced eGFR in patients with type 2 diabetes, even in early stages of the disease [38]. Additionally, this vascular lesion has been strongly linked to an elevated risk of cardiovascular events in individuals with DN [39]. Interstitial and vascular lesions in DN according to RPS classification are presented in Table 2.

## 4. Clinical Course

### 4.1. Glomerular Hyperfiltration

Glomerular hyperfiltration represents an early and potentially reversible stage of renal dysfunction that occurs prior to the onset of albuminuria and reflects hemodynamic alterations induced by hyperglycemia [40,41]. It can be assessed using estimated glomerular filtration rate (eGFR) derived from serum creatinine-based equations, or more accurately by measured GFR using exogenous markers such as inulin, iohexol, or iothalamate. Hyperfiltration may also be inferred from an increased filtration fraction reflecting the relationship between GFR and renal plasma flow (RPF). It is generally defined as a GFR above ~130–140 mL/min/1.73 m^2^, although thresholds vary across studies [42].

This phenomenon is associated with increased risk of subsequent nephropathy, structural kidney damage, and adverse clinical outcomes, including progression to ESRD [41]. Elevated GFR in early diabetes may therefore serve as an important marker for identifying high-risk individuals, particularly in prediabetic states, where early preventive strategies may be most effective [40,41].

### 4.2. Clinical Phenotypes and the Role of Proteinuria in DKD

DKD is traditionally defined by persistent albuminuria followed by a progressive decline in glomerular filtration rate (GFR), representing the classical clinical phenotype, commonly observed in patients with persistent metabolic abnormalities and poor glycemic control [43]. Non-proteinuric DKD appears to be more common than previously recognized, accounting for approximately 20% of cases in individuals with type 1 DM [44] and up to 50% in those with type 2 DM [45]. This phenotype is characterised by progressive decline in kidney function without significant albuminuria and, compared with albuminuric DKD, is generally associated with a slower rate of GFR decline and may involve a greater contribution of vascular and tubulointerstitial injury. Reported associations include female sex, hypertension, smoking, absence of diabetic retinopathy, and previous RAAS inhibitor therapy [43]. Other proposed non-classical clinical trajectories include albuminuria regression phenotype (may be associated with improved glycemic and blood pressure control and the use of renoprotective therapies, including RAAS inhibitors and SGLT2 inhibitors) and rapid GFR decline phenotype (defined as a loss of ≥5 mL/min/1.73 m^2^/yr; may occur with or without concomitant albuminuria and is associated with an increased risk of kidney failure) [43].

Despite the heterogeneity of DKD, albuminuria remains its key clinical hallmark [46], while proteinuria not only reflects glomerular injury but also contributes directly to disease progression and serves as an indicator of glomerular damage severity [47]. Importantly, different protein components in urine may correspond to distinct pathological processes, as increased excretion of macromolecular proteins reflects disruption of the glomerular filtration barrier [20]. Patients with proteinuria generally exhibit more advanced structural lesions and impaired renal function, although a substantial proportion may still present with relatively mild histological changes [48]. Given that pathological severity may correlate with renal prognosis, early identification of DKD—even in normoalbuminuric individuals—may allow timely therapeutic intervention and delay disease progression [46].

As kidney disease progresses beyond glomerular involvement, tubulointerstitial injury also warrants consideration. In advanced tubular dysfunction, impaired distal tubular potassium and hydrogen ion secretion may lead to hyperkalemia and mild metabolic acidosis, particularly in patients with hyporeninemic hypoaldosteronism (type IV renal tubular acidosis) [49].

## 5. Renal Biopsy in DKD

### 5.1. Indications for Kidney Biopsy in Patients with Diabetes

In clinical practice, DKD is conventionally diagnosed based on persistent albuminuria, progressive eGFR decline, and the presence of diabetic retinopathy, without recourse to histological confirmation [50]. However, biopsy-based studies and meta-analyses have demonstrated that NDKD (whether isolated or superimposed upon DKD) accounts for a substantial proportion of cases in this population, underscoring the limitations of clinical diagnosis alone [51,52]. Neither KDIGO nor ADA guidelines define rigid biopsy criteria, but both endorse their consideration when histological diagnosis is expected to influence management [50,53]. Recognised indications include rapid or disproportionate renal function decline, acute-onset nephrotic-range proteinuria, rapidly rising or high proteinuria, active urinary sediment, absence of retinopathy, short diabetes duration (<5 yr), AKI of unclear etiology, suspected presence of systemic disease, and chronically well-controlled blood glucose levels [51,52,54,55,56]. In the absence of dedicated European guidelines, clinical decision-making remains guided by KDIGO recommendations, with emphasis on individualized assessment of diagnostic uncertainty and therapeutic impact [50,56].

### 5.2. Diagnostic Value of Renal Biopsy and Clinical-Pathological Correlations in DN

Renal biopsy plays a central role in evaluating both the nature and severity of kidney injury in patients with DKD [57]. It remains the gold standard for diagnosing DN, particularly in patients with type 2 diabetes mellitus presenting atypical clinical features, and early biopsy assessment may facilitate individualized treatment strategies [58].

The use of the Tervaert classification system improves inter-observer reproducibility by providing a standardized framework for histopathological evaluation [59].

Although the RPS classification is a valuable tool for histopathological assessment, its prognostic utility remains uncertain, with inconsistent findings across different populations. Studies from Asian cohorts have reported associations between RPS classes, glomerular and tubulointerstitial lesions, and renal outcomes [60,61,62,63]. However, other reports have indicated that only tubulointerstitial lesions, but not glomerular lesions, were independently associated with renal prognosis [64]. European cohorts have shown limited prognostic value of RPS classes, with IFTA score and GBM thickness demonstrating stronger associations with renal survival [65]. Moreover, correlations between RPS classes and laboratory parameters, including albuminuria and proteinuria, appear to be weak [48]. Nevertheless, recent studies support the clinical relevance of RPS classification, although its prognostic value remains context-dependent and should be interpreted together with tubulointerstitial and vascular lesions and additional biomarkers [17,66].

Clinical parameters consistently lack sufficient discriminatory accuracy to distinguish DN from NDKD [51], a limitation of relevance where therapeutic strategies diverge substantially (as in immune-mediated glomerulonephritis requiring immunosuppression). Importantly, biopsy identification of NDKD or mixed DN/NDKD carries distinct prognostic and therapeutic implications. Cohort data suggest more favourable renal survival in appropriately managed NDKD patients compared to pure DKD [51,52]. Superimposed acute lesions such as acute tubular injury or thrombotic microangiopathy may identify patients at risk of abrupt renal function decline amenable to targeted intervention.

Although imaging techniques can identify certain urological and macrovascular complications, definitive differential diagnosis in many cases still relies on kidney biopsy [67]. Histopathological studies have advanced understanding of renal damage mechanisms, enabling identification of diverse injury phenotypes and informing individualized treatment strategies [68]. Integration of histopathological, omics, and imaging findings further optimizes patient-specific management [68].

Given that DKD is the leading cause of end-stage renal disease worldwide and exhibits heterogeneous progression, accurate prediction of disease onset and trajectory remains essential for early intervention and personalized care [69].

### 5.3. Limitations of Kidney Biopsy: Advances in Imaging and Biopsy Techniques

Kidney biopsy carries several well-recognized limitations, including procedural risks such as hemorrhage and arteriovenous fistula formation [52]. However, the risk of complications associated with renal biopsy in individuals with diabetes mellitus is not higher than in the general population [70]. Moreover, obesity and diabetic kidney disease have been identified as factors associated with a lower risk of significant post-biopsy bleeding [71]. Other limitations include sampling variability arising from the focal and heterogeneous distribution of diabetic lesions [10]; inter-observer variability in histopathological interpretation even under standardized classification systems [10,21]; and its inherently static nature, which precludes assessment of disease evolution over time. Access to expert renal pathology services remains uneven across healthcare settings, further limiting its broad applicability.

Parallel technological innovations have substantially improved both the procedural safety and the diagnostic yield of percutaneous kidney biopsy. Contemporary real-time ultrasound-guided biopsy techniques have significantly reduced rates of hemorrhagic complications, while the standardization of spring-loaded automated biopsy devices has improved the consistency of core tissue procurement [72].

Emerging optical imaging modalities, including multiphoton microscopy (MPM) and optical coherence tomography (OCT), offer high-resolution, label-free visualization of renal microarchitecture and are under active investigation as tools for minimally invasive or potentially non-invasive assessment of kidney pathology. MPM is widely used in preclinical renal research to study dynamic physiological processes, including capillary blood flow, glomerular function, vascular permeability, and proximal tubular transport, as well as to investigate disease mechanisms and therapeutic responses [73]. OCT, originally established in ophthalmology and more recently applied in fields such as dermatology, gastroenterology, and urology, has also demonstrated potential for renal tissue imaging by enabling rapid cross-sectional visualization of major structural compartments [74]. Although both techniques remain largely investigational in nephrology, they offer promising, potentially complementary roles in improving the structural and functional assessment of renal pathology beyond conventional biopsy.

## 6. Recent Advances and Future Directions in Kidney Biopsy for Diabetic Patients

### 6.1. Artificial Intelligence and Digital Pathology

One of the most transformative advances in the field of renal pathology is the integration of digital pathology platforms and artificial intelligence (AI) into the analytical pipeline for kidney biopsy specimens. Whole-slide imaging (WSI) technology enables complete digitization of biopsy slides, generating high-resolution datasets enabling both computational analysis and systematic large-scale archiving [75].

Deep learning algorithms, in particular convolutional neural networks (CNNs), have demonstrated high accuracy in the automated identification and segmentation of key renal microstructures, including glomeruli, proximal and distal tubules, and interstitial compartments [76]. Advanced architectures such as panoptic segmentation networks allow simultaneous instance detection and semantic classification of multiple histological features within a single image field, substantially exceeding the throughput and reproducibility achievable by manual assessment [77].

The principal advantages of AI-assisted pathology over conventional visual scoring include objective, quantitative measurement of histological lesions; significant reduction in inter-observer and intra-observer variability; high-throughput analysis enabling population-scale studies; and enhanced reproducibility facilitating multicentre standardization [76,78]. In the context of biopsy-evaluated DKD, particularly DN, AI models are currently being developed and validated to: (i) differentiate biopsy-proven DN from NDKD and mixed forms; (ii) quantify tubulointerstitial fibrosis and inflammatory infiltration; and (iii) generate histology-based outcome predictions [79,80].

Preliminary evidence suggests that AI-driven scoring systems may surpass conventional semi-quantitative classification schemes in predicting disease trajectory and the rate of renal function decline [78]. However, prospective external validation across ethnically and geographically diverse cohorts remains a prerequisite before integration into routine clinical workflows [81].

### 6.2. Integration of Multi-Omics Technologies

The application of multi-omics technologies to kidney biopsy tissue has emerged as one of the most promising frontiers in modern nephrology research. Multi-omics is an umbrella term encompassing several complementary molecular profiling techniques including transcriptomics (the study of gene activity), proteomics (the study of proteins), metabolomics (the study of metabolic products), and epigenomics (the study of heritable changes in gene expression beyond DNA sequence). Together they allow researchers to characterize the biological pathways driving diabetic kidney injury with a level of detail that was previously unattainable.

By applying these techniques to specific tissue compartments within the kidney biopsy, it becomes possible to distinguish between disease processes occurring in the glomeruli versus the tubulointerstitium. This compartment-specific approach provides insight into how different cell types, including podocytes, each contribute to kidney injury in DKD. Bulk transcriptomic studies, which measure overall gene expression across many cells simultaneously, have identified molecular signatures associated with well-established pathological processes in DN, including inflammation, pro-fibrotic signaling, oxidative stress, and metabolic dysregulation with representative biomarkers such as upregulation of the inflammatory chemokine CCL2 (MCP-1), increased expression of extracellular matrix-related genes, and downregulation of podocyte-associated genes. Although these findings have substantially advanced mechanistic understanding of DN, their clinical application remains largely confined to the translational research setting [82]. More recently, single-cell RNA sequencing (scRNA-seq) revealed a previously unrecognized degree of cellular diversity within podocyte, proximal tubular epithelial, and immune cell populations in DN [83]. This cell-level resolution has uncovered distinct subpopulations of cells with differing transcriptional responses to chronic high glucose exposure, offering a more granular understanding of disease heterogeneity. However, despite its considerable research value, routine clinical implementation of scRNA-seq remains constrained by technical complexity, cost, and a lack of methodological standardization. Complementing this, spatial transcriptomics combines positional information with molecular profiling, allowing gene expression data to be mapped directly onto tissue architecture [84]. This approach effectively bridges the longstanding conceptual gap between traditional histopathology and molecular genomics and carries considerable translational potential by enabling direct correlation of molecular signatures with histological lesions, although its clinical application remains largely investigational at present [85].

At the protein level, proteomic analyses of microdissected glomerular and tubulointerstitial compartments have identified dysregulated protein networks involved in basement membrane remodeling, mitochondrial dysfunction, and complement pathway activation. All of which are implicated in the progression of DN. Candidate protein biomarkers identified through these approaches include complement-related proteins and kidney injury molecule-1 (KIM-1), although none have yet been incorporated into routine biopsy assessment [86,87]. Taken together, these converging multi-omics findings are increasingly informing the development of molecular subclassifications of DKD that may complement and refine existing histopathological grading systems, with the goal of improving prognostic stratification and guiding more personalized therapeutic strategies [82,83]. Nevertheless, further prospective validation in larger, well-characterized cohorts is required before these technologies can be routinely incorporated into clinical decision-making.

### 6.3. Non-Invasive Biomarkers and the Concept of “Virtual Biopsy”

In recognition of the invasive nature of kidney biopsy and the associated procedural risks, considerable research effort has been directed towards developing non-invasive diagnostic surrogates capable of replicating histopathological information. Candidate biomarkers derived from urine and plasma, encompassing structural proteins, cytokines, chemokines, microRNAs, and extracellular vesicles, are being systematically investigated as potential surrogates for specific histological lesions. Among the most extensively studied are kidney injury molecule-1 (KIM-1) and β2-microglobulin, which have shown promise for predicting disease progression and tubular injury but do not reliably reproduce biopsy findings [86].

Machine learning models integrating biomarker profiles with clinical and demographic data have given rise to the concept of the “virtual biopsy”—a computational framework aiming to predict histological diagnoses and lesion severity without recourse to tissue sampling [87]. While conceptually appealing, current virtual biopsy approaches lack the diagnostic accuracy required to supplant conventional histopathological assessment in clinical practice. Current models appear most useful for identifying patients at increased risk of advanced DKD or rapid renal function decline, rather than for establishing a definitive histological diagnosis. Their performance remains insufficient to reliably distinguish biopsy-proven DN from NDKD or mixed pathological forms, limiting their applicability in patients with atypical clinical presentations, in whom conventional kidney biopsy remains indispensable [51,86]. Nevertheless, they promise stratification tools for identifying patients at elevated risk of NDKD or rapid renal function deterioration, thereby rationalizing the utilization of invasive biopsy resources.

### 6.4. Future Perspectives and Challenges

Despite the substantial progress outlined above, significant challenges persist in translating these advances into routine clinical practice. In particular, technologies such as single-cell and spatial transcriptomics, proteomic profiling, and virtual biopsy currently remain predominantly research tools, and their clinical implementation will require demonstration of incremental diagnostic or prognostic value beyond conventional histopathological assessment in prospective multicentre studies [84,85]. The implementation of AI-assisted digital pathology and multi-omics profiling requires institutional standardization, rigorous prospective validation, and seamless integration into existing clinical informatics workflows. Ensuring data interoperability between institutions and establishing open, curated biobanks of annotated biopsy specimens are prerequisite conditions for the development of robust, generalizable predictive models.

Cost, technical complexity, and the requirement for specialized infrastructure may limit equitable adoption of advanced molecular diagnostic approaches, particularly in resource-constrained healthcare environments [88]. Ethical considerations pertaining to data privacy, algorithmic transparency, and regulatory frameworks governing AI-based diagnostic tools must be systematically addressed prior to widespread clinical deployment [89].

In the foreseeable future, kidney biopsy is poised to evolve into a central pillar of a multimodal precision medicine framework for clinically defined DKD and biopsy-confirmed DN, in which histopathological assessment, digital imaging, molecular profiling, and longitudinal clinical data are integrated to generate individualized diagnostic, prognostic, and therapeutic recommendations [90]. Realizing this potential will require sustained interdisciplinary collaboration among nephropathologists, clinical nephrologists, computational biologists, and bioinformaticians.

## 7. Conclusions

Kidney biopsy remains a cornerstone of precision diagnosis and prognostic stratification in DKD, providing critical insights into the structural heterogeneity of renal injury driven by hyperglycemia-induced metabolic, inflammatory, and fibrotic pathways. Histopathological classification, particularly the RPS/Tervaert system, together with assessment of tubulointerstitial and vascular lesions, provides important prognostic information and complements clinical parameters such as proteinuria and eGFR decline. However, given procedural risks, sampling variability, and the heterogeneous nature of DKD, biopsy should be applied selectively, particularly in patients with atypical clinical presentations, including rapid loss of kidney function, non-albuminuric kidney disease, or suspected non-diabetic kidney disease or mixed renal pathology.

Advances in imaging techniques, artificial intelligence-assisted digital pathology, and multi-omics approaches are expected to enhance the diagnostic precision, reproducibility, and prognostic value of kidney biopsy rather than replace tissue-based evaluation. Emerging non-invasive biomarkers and “virtual biopsy” models may provide additional tools for risk assessment and disease monitoring, although their current role remains complementary. Future research should focus on harmonizing pathological scoring systems, developing and validating multicenter AI-based models, and identifying highly specific non-invasive biomarkers. Ultimately, kidney biopsy is evolving from a purely diagnostic procedure into an integral component of a multimodal precision medicine framework, integrating histopathological, molecular, and computational data to improve individualized risk prediction and guide targeted therapeutic strategies in DKD.

## Figures and Tables

**Table 1:** Glomerular classes of DN according to the RPS classification *.

Class	Histopathological Features		
Class I	Mild or nonspecific light microscopic changes with electron microscopy–confirmed glomerular basement membrane thickening		
Class IIa	Mild mesangial expansion involving >25% of the mesangial areas examined		
Class IIb	Severe mesangial expansion involving >25% of the mesangial areas examined		
Class III	Nodular sclerosis with at least one Kimmelstiel–Wilson lesion		
Class IV	Advanced diabetic glomerulosclerosis with >50% global glomerulosclerosis		

* Higher RPS classes indicate more advanced glomerular lesions and may be associated with poorer renal outcomes; however, prognostic interpretation requires integration with tubulointerstitial and vascular changes and clinical parameters (see main text for details).

**Table 2:** Interstitial and vascular lesions in DN according to RPS classification *.

Lesion	Criteria	Score
Interstitial fibrosis and tubular atrophy (IFTA)	No IFTA	0
	IFTA involving <25% of cortical area	1
	IFTA involving 25–50% of cortical area	2
	IFTA involving >50% of cortical area	3
Interstitial inflammation	Absent	0
	Inflammation limited to areas of IFTA	1
	Inflammation present in areas without IFTA	2
Arteriolar hyalinosis	Absent	0
	One area of arteriolar hyalinosis	1
	More than one area of arteriolar hyalinosis	2
Arteriosclerosis	No intimal thickening	0
	Intimal thickening less than medial thickness	1
	Intimal thickening greater than medial thickness	2

* Interstitial and vascular lesions are important determinants of renal prognosis in diabetic nephropathy. Although the RPS classification primarily focuses on glomerular lesions, tubulointerstitial and vascular changes—particularly interstitial fibrosis and tubular atrophy (IFTA)—have shown strong associations with renal function decline and renal survival.

## Data Availability

No new data were created or analyzed in this study.

## Abbreviations

The following abbreviations are used in this manuscript:

ADA	American Diabetes Association
AI	Artificial Intelligence
AGEs	Advanced Glycation End-Products
CKD	Chronic Kidney Disease
CNN	Convolutional Neural Network
DKD	Diabetic Kidney Disease
DM	Diabetes mellitus
DN	Diabetic nephropathy
eGFR	Estimated Glomerular Filtration Rate
ESRD	End-Stage Renal Disease
GBM	Glomerular Basement Membrane
IFTA	Interstitial Fibrosis and Tubular Atrophy
KDIGO	Kidney Disease Improving Global Outcomes
MPM	Multiphoton Microscopy
OCT	Optical Coherence Tomography
NAG	N-Acetyl-*β*-D-Glucosaminidase
NDKD	Non-Diabetic Kidney Disease
PKC	Protein Kinase C
RAAS	Renin–Angiotensin–Aldosterone System
RAGE	Receptor for Advanced Glycation End-Products
RBP	Retinol-Binding Protein
ROS	Reactive Oxygen Species
RPS	Renal Pathology Society
scRNA-seq	single-cell RNA sequencing
SHG	Second Harmonic Generation
TGF-β	Transforming Growth Factor-β
TPEF	Two-Photon Excited Fluorescence
WSI	Whole-Slide Imaging
